# Social prescribing for anxiety and depression in older adults: mechanistic pathways, implementation requirements, and limitations of current evidence

**DOI:** 10.3389/fpubh.2026.1958834

**Published:** 2026-09-29

**Authors:** Bo Yang, Bang He, Yang Gao, Hong Chen, Xiaofan Yuan

**Affiliations:** 1 Sichuan Provincial People's Hospital, School of Medicine, University of Electronic Science and Technology of China, Chengdu, China; 2 Xindu District People's Hospital of Chengdu, Chengdu, China

**Keywords:** anxiety, depression, implementation science, older adults, social determinants of health, social prescribing

## Abstract

Population ageing and the rising burden of multimorbidity require late-life anxiety and depression to be addressed beyond symptom control. Loneliness, social isolation, restricted activity, and loss of meaningful roles frequently interact with physical illness and functional decline; however, these determinants are not readily modified through conventional clinical care alone. Social prescribing offers a range of supported pathways through which health and care professionals connect individuals with non-clinical community resources, ranging from light-touch referrals to more holistic, relationally supported models. This narrative review used a structured literature search and an explicit evidence framework to distinguish between direct evaluations of social prescribing services, pathway-level evidence on constituent activities and mechanisms, and implementation evidence concerning referral, connector support, and community capacity. Current findings suggest the potential benefits for loneliness, social participation, quality of life, and selected depressive outcomes through strengthened social connections, behavioural activation, physical activity, engagement with nature and culture, and the restoration of valued social identities. Nevertheless, high-quality trials specifically enrolling older adults with diagnosed anxiety or depressive disorders remain scarce. Definitions of social prescribing, intervention intensity, comparators, and outcome measures vary substantially, while several proposed mechanisms are supported by indirect evidence rather than formal mediation analyses. Effectiveness, therefore, depends not only on the activity offered but also on appropriate risk stratification, shared decision-making, sufficient relational support when required, accessible community resources, and closed-loop evaluation. Future research should use complex intervention and implementation science frameworks to identify appropriate populations, core and adaptable components, causal mediators, cost-effectiveness, and equitable models that can be embedded within local primary care and ageing service systems.

## 1 Introduction

Population ageing is accelerating worldwide. The World Health Organization estimates that the number of people aged 60 years or older will reach approximately 2.1 billion by 2050, with most living in low- and middle-income countries (1). Later life is often accompanied by chronic physical illness, functional limitation, cognitive change, retirement, bereavement, and the contraction of social networks. These experiences create a context in which anxiety and depression are frequently multifactorial, under-recognised, and closely intertwined with multimorbidity. Systematic reviews show marked geographical variation in depressive, anxiety, and stress symptoms among older adults (2), with a particularly high burden in low- and middle-income settings (3). Late-life depression is also associated with multimorbidity, biological ageing, functional deterioration, and increased mortality (4–6). Its consequences therefore extend beyond emotional symptoms to affect independence, chronic disease self-management, and the use of health and social care services.

Services for older adults with mental health problems remain organised largely around diagnosis, pharmacotherapy, and specialist care. These approaches are indispensable, particularly for moderate-to-severe disorders, but they do not readily modify the social circumstances that sustain distress. Among older adults with mild-to-moderate symptoms, pronounced social adversity, or reluctance to seek specialist mental health care, loneliness, poverty, caregiving strain, transport barriers, and loss of valued roles may remain largely unaddressed. Stigma, cognitive changes, and concerns about adverse effects may further reduce help-seeking and treatment continuity (7). Social prescribing has attracted growing attention in response to this gap. At its broadest, it involves identifying non-medical needs and connecting people with community-based resources and services (8, 9). Importantly, social prescribing is not a single uniform model. Kimberlee described a continuum ranging from signposting to light, medium, and holistic approaches, with increasing intensity of assessment, coordination, and relational support (10). More recent consensus research similarly indicates that a dedicated connector or link worker is not mandatory in every model (11). In this review, the term social prescribing therefore encompasses light, medium, and holistic supported referral models, while simple signposting is treated as the least intensive form at the boundary of social prescribing rather than being assumed to represent the entirety of social prescribing. Where a link worker or equivalent connector is discussed, this refers to models in which such a role is actually present. Social prescribing should not replace evidence-based pharmacological or psychological treatment; rather, it offers a complementary service pathway for addressing social determinants that conventional healthcare is poorly equipped to address directly (12).

Assertions that social prescribing is effective require careful qualification. The literature combines at least three distinct forms of evidence: Direct evaluations of a social prescribing service; pathway-level studies of constituent activities such as exercise, nature engagement, arts participation, or volunteering; and implementation research concerning connectors/link workers, referral processes, and community resources. These bodies of evidence answer different questions. Without explicit separation, the effect of a single community activity may be attributed to the entire social prescribing model, and observational associations may be misinterpreted as causal effects. This review therefore uses a narrative synthesis to examine the conceptual boundaries of social prescribing, its plausible pathways of action, the conditions required for implementation, and the limitations of current evidence for anxiety and depression in older adults, while explicitly distinguishing direct evidence of social prescribing from indirect evidence supporting its mechanistic plausibility.

## 2 Methods

### 2.1 Review design and literature search

This narrative review used a structured literature search to identify evidence relevant to social prescribing for anxiety and depression in older adults. PubMed was searched from database inception to 23 August 2026. The bibliographic search was supplemented by screening the reference list of relevant systematic reviews and key articles, together with targeted searches of authoritative guidance and policy documents from organisations including the World Health Organization, the UK National Institute for Health and Care Excellence (NICE), and the Medical Research Council.

The main PubMed search combined terms relating to social prescribing, older populations, and mental health outcomes: (“social prescribing” OR “social prescription” OR “link worker*” OR “community referral*”) AND (“older adult*” OR “older people” OR aged OR elderly) AND (depress* OR anxi* OR loneliness OR “social isolation” OR wellbeing OR “well-being”). Additional searches incorporated terms relating to implementation and proposed intervention pathways, including behavioural activation, physical activity, nature-based activities, arts and cultural participation, volunteering, nutrition, digital support, and community capacity. Reference lists of relevant reviews and included articles were also examined to identify additional sources pertinent to the conceptual, mechanistic, and implementation questions addressed in this review.

### 2.2 Eligibility, scope, and evidence types

For direct evidence, studies were considered relevant when participants entered an explicitly described social prescribing or social prescription pathway through healthcare, social care, or closely linked community services. Older adults were operationalised as populations aged 60 years or older or as study populations with a reported mean or median age of at least 60 years. Studies involving broader adult populations were considered when they provided directly relevant evidence on implementation, mechanisms, or service organisation and when older adult-specific evidence was limited.

Eligible evidence included systematic reviews and meta-analyses, randomised and quasi-randomised trials, observational studies, qualitative and mixed methods research, implementation studies, relevant clinical and public health guidance, and foundational theoretical literature. Paediatric studies, interventions confined to conventional clinical treatment without a non-clinical community component, duplicate reports that did not contribute additional relevant information, and publications unrelated to the review questions were excluded. The review was restricted to English-language publications.

### 2.3 Evidence classification and narrative synthesis

Evidence was organised into three analytical layers according to its relationship to social prescribing. Direct evidence comprised studies evaluating an identifiable social prescribing service or pathway and reporting health, psychosocial, functional, or service outcomes. Pathway evidence comprised studies examining constituent activities or plausible mechanisms—including social connection, behavioural activation, physical activity, nature exposure, arts and cultural participation, volunteering, and dietary or lifestyle factors—even when these components were evaluated outside a complete social prescribing service. Implementation evidence addressed referral processes, connector or link worker functions, community capacity, engagement, digital support, equity, and evaluation systems.

An iterative thematic narrative synthesis was used to compare findings across these evidence layers and to identify recurrent mechanisms, implementation conditions, and areas of uncertainty. Particular attention was given to distinguishing evidence arising from the evaluation of a complete social prescribing pathway from evidence supporting the plausibility of individual activities or underlying mechanisms. Differences in interpretation were discussed among the authors and resolved through consensus.

Table 1 represents an author-developed synthesis of proposed pathways, relevant evidence, and principal evidential limitations. Table 2 was similarly developed from the reviewed social prescribing and implementation literature, with its domains informed by concepts from the Medical Research Council guidance for complex interventions, Reach Effectiveness Adoption Implementation Maintenance (RE-AIM), and the Consolidated Framework for Implementation Research (CFIR) (13–15). The indicators presented in Table 2 are intended as a pragmatic framework for evaluation rather than as a validated measurement instrument.

**Table 1:** Proposed pathways linking social prescribing to anxiety and depression in older adults and their evidential limits.

Pathway	Typical resources or services	Potential outcomes	Current evidence	Principal limitations
Social connection and belonging	Peer support groups, cultural activities, community hubs, and volunteering	Loneliness, social support, wellbeing, and depressive symptoms	Direct and pathway evidence shows encouraging signals (20, 22–25, 50)	Loneliness is often conflated with clinical depression; self-selection and the quality of social ties complicate causal attribution (20, 22–25, 50).
Behavioural activation and self-efficacy	Supported referral, value-aligned activities, and peer companionship	Avoidance, activity, self-efficacy, and emotional wellbeing	Theoretical and indirect evidence is relatively strong (26–31)	Social prescribing is not equivalent to formal behavioural activation therapy; mediation analyses and component-level causal tests are scarce (28, 29, 36).
Physical activity	Walking, tai chi, dance, group exercise, and strength and balance programmes	Depressive symptoms, function, activity, and quality of life	Pathway evidence is substantial, but direct social prescribing evidence is heterogeneous (32–35, 37, 38)	Dose, co-interventions, selection effects, and biological mechanisms have not been adequately separated (32, 33, 35, 37, 38).
Nature, arts, and cultural participation	Gardening, nature walks, singing, art-making, and museum programmes	Mood, cognition, meaning, loneliness, and quality of life	Systematic reviews support potential benefit (40–43, 47–50)	Effects of nature, movement, social interaction, staff attention, and cultural participation overlap, limiting attribution to a single component (40–43, 47–50).
Nutrition and lifestyle	Nutrition counselling, cooking groups, and shared meals	Diet quality, anxiety and depressive symptoms, and social interaction	Evidence is mainly observational or mechanistic (51–54).	High-quality trials evaluating a complete social prescribing pathway are lacking; nutrition is best treated as an adjunct lifestyle component rather than a core mechanism (51–54).

**Table 2:** Core domains for the implementation and evaluation of social prescribing for older adults.

Implementation domain	Core question	Suggested indicators
Identification and stratification	Who is suitable for social prescribing, and who requires priority specialist treatment?	Anxiety/depression severity, suicide risk, loneliness, function, cognition, transport and financial barriers (13, 55, 56, 59)
Shared decision-making and matching	Does the option fit the person’s values, culture, interests, and abilities?	Goal concordance, involvement in choice, first-contact rate, refusal and withdrawal reasons (13, 60–63)
Connector/link worker support (where applicable)	Is the intensity of navigation/support sufficient to overcome barriers to participation?	Number of contacts, accompanied referral, problem-solving, timely escalation to clinical services (60–63)
Community capacity	Are resources accessible, affordable, and able to accommodate referrals?	Coverage, waiting time, cost, accessibility, programme capacity, burden on community organisations (14, 15, 64–67)
Sustained engagement	Does referral translate into meaningful and continuing participation?	Actual uptake, duration, relationship quality, goal attainment, experience, adverse events (13, 14, 63, 69)
Outcomes and equity	Who benefits, who is missed, and are effects sustained?	Anxiety, depression, loneliness, quality of life, function, service use, stratified equity, cost outcomes (14, 15, 65, 71)

The suggested indicators in this table are author-proposed and informed by the reviewed social prescribing literature together with MRC complex intervention guidance, RE-AIM, and the CFIR (13–15). They are intended as a pragmatic evaluation set, not as a validated scale or mandatory framework.

### 2.4 Appraisal and weighting of evidence

Given the heterogeneity of the included evidence, methodological appraisal was undertaken qualitatively rather than through a single risk-of-bias instrument applied across all source types. Evidence was considered in relation to study design, sample size, comparator characteristics where applicable, potential selection and attrition bias, duration of follow-up, consistency with other findings, directness to older adults and to social prescribing pathways, and relevance to the specific claim being examined.

Greater inferential weight was assigned to systematic reviews, meta-analyses, and controlled trials when considering intervention effectiveness. Qualitative, realist, and implementation studies were used primarily to inform the understanding of acceptability, participant experience, mechanisms, contextual influences, and implementation processes. Observational and theoretical studies were used principally to support associations or mechanistic plausibility and were not interpreted as establishing the causal effects of social prescribing. Claims based on indirect, mechanistic, observational, or inconsistent evidence were therefore interpreted cautiously and identified as such in the synthesis.

The Scale for the Assessment of Narrative Review Articles (SANRA) was used to inform the overall reporting, scientific reasoning, and presentation of the review (16).

### 2.5 Author positionality

The author team is based in hospital and medical school settings in China, with perspectives grounded primarily in clinical care, ageing, and health services research. These backgrounds informed the review’s attention to clinical risk stratification, the integration of social prescribing with established pharmacological and psychological care, and its adaptation to local health and community service systems. To incorporate perspectives beyond healthcare settings, the synthesis also drew on qualitative, community sector, social care, and implementation research, and context-dependent findings were interpreted, with attention given to differences in service organisation, community resources, and population needs.

## 3 Conceptual boundaries and an evidence framework

Social prescribing is best understood as a way of organising care rather than as a single standardised treatment. Programmes vary in intensity. In Kimberlee’s typology, signposting directs a person toward available resources; light models refer people to a specific programme for a defined need; medium models add a connector or link worker conversation and tailored referral; and holistic models provide broader person-centred assessment, co-produced planning, supported access, and follow-up (10). Contemporary consensus definitions similarly caution against assuming that every social prescribing model contains a dedicated connector (11). Accordingly, the core functions relevant to this review are identifying non-medical needs, matching individuals to community assets via a person-centred approach, and providing supported connections proportionate to need. Shared decision-making, a dedicated connector, and sustained follow-up are particularly characteristic of medium and holistic models (8, 9, 17, 18). The intervention is therefore not synonymous with the community activity itself, and the intensity of navigation/support should be reported explicitly when interpreting outcomes.

Evidence relevant to mental health in later life can be organised into three layers. Direct evidence evaluates people who enter a social prescribing pathway through primary care or another health or care service and measures outcomes such as anxiety, depression, loneliness, quality of life, or service use. Pathway evidence addresses whether social connection, behavioural activation, physical activity, nature exposure, or cultural participation can influence mental health when these components are studied outside a complete social prescribing service. In this study, a ‘complete social prescribing pathway’ refers to a programme in which identification or referral, connection or navigation, participation in a community resource, and—where the model specifies it—follow-up are evaluated as an integrated service process. It does not indicate that every participant should complete every offered activity, nor does it imply that all legitimate social prescribing models require long-term holistic support. Implementation evidence examines how referral processes, connector/link worker capabilities, community infrastructure, and evaluation systems shape feasibility, acceptability, and sustainability. Direct evidence estimates intervention effects, pathway evidence supports mechanistic explanations, and implementation evidence identifies the conditions under which benefits might occur; however, none can substitute for the others.

This distinction also clarifies the clinical boundaries of social prescribing. Severe or clinically complex depression, acute suicide risk, psychotic symptoms, mania, and complex or treatment-refractory anxiety disorders require timely clinical assessment and established treatment; social prescribing must not delay either. Its clearest role may be as an adjunct for older adults with mild-to-moderate symptoms, prominent loneliness or social isolation, coexisting chronic disease and functional difficulties, or unmet social needs despite routine care (18–20). The appropriate question is therefore not simply whether social prescribing works, but for whom it is suitable, at what intensity, through which components, and under what service and community conditions. Figure 1 summarises this complex intervention logic and explicitly distinguishes the target population, service functions, community resources, proposed mediators, contextual modifiers, implementation outcomes, and safety escalation.

**Figure 1:**
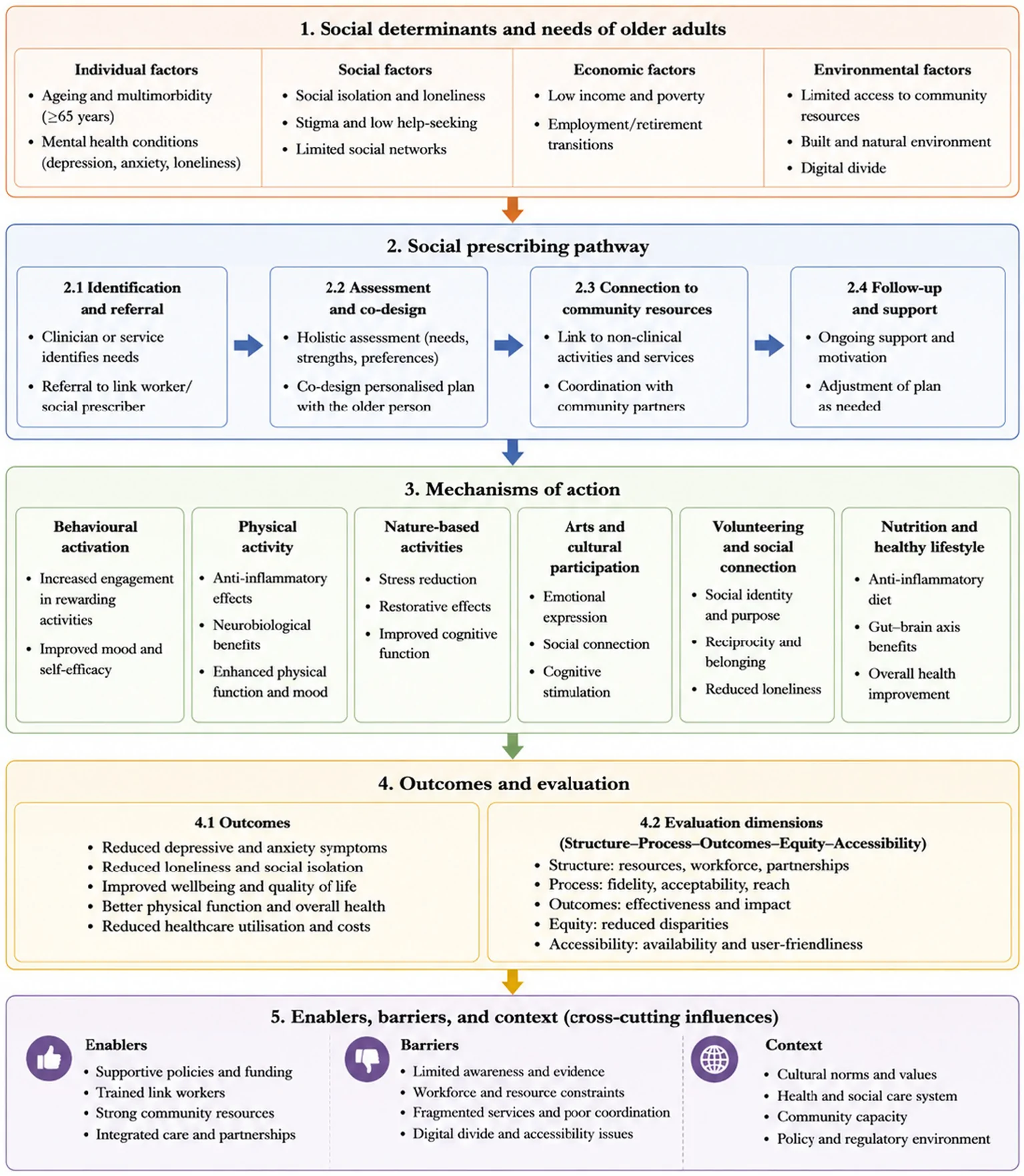
Conceptual framework of social prescribing for promoting mental health in older adults. Social prescribing addresses social determinants and connects older adults with community resources through a structured pathway. Multiple mechanisms of action contribute to improved mental health and wellbeing. Outcomes are evaluated across structure, process, outcomes, equity, and accessibility, with enablers, barriers, and context influencing each stage (13–15).

## 4 Pathways through which social prescribing may influence anxiety and depression

### 4.1 Social connection: from contact to belonging

Loneliness is the subjective experience that the quantity or quality of one’s relationships falls short of what is desired, whereas social isolation refers primarily to an objective lack of social contact. The two are related but not interchangeable. The World Health Organization recognises social isolation and loneliness as important public health concerns (21). Social prescribing may increase opportunities for sustained interaction through peer groups, cultural programmes, volunteering, and other community activities. However, greater contact does not automatically relieve loneliness. Alignment with personal interests, continuity of attendance, reciprocity, psychological safety, and the ability to exercise choice may matter more than the frequency of activities alone (20, 22–24).

A Canadian study found that emotional and social loneliness differed in their associations with wellbeing and openness to discussing health needs, suggesting that the uniform referral pathways are unlikely to meet heterogeneous needs (22). Older participants in a Portuguese study described leaving the house, talking with others, and laughing together as salient benefits of social prescribing (23). Research on community hubs similarly highlighted familiar settings, supportive staff, and reciprocity among members as factors sustaining participation (24). A 2024 systematic review of UK social prescribing initiatives targeting social connection further suggested that the quality of social ties, shared identity, perceived support, and self-efficacy may be more important than simply increasing the contact frequency (25). These findings suggest that the aim is not merely to place an older person in a group but to facilitate entry into a social setting in which trust, mutuality, and a valued identity can develop. However, the evidence should not be overinterpreted. Many studies enrol people at risk of loneliness or isolation rather than patients with clinically diagnosed anxiety or depressive disorders, prioritise loneliness or wellbeing as outcomes, and use observational, qualitative, or small-scale designs. Social connection is therefore a credible intervention target; however, current evidence does not establish the efficacy of social prescribing for anxiety or depressive disorders.

### 4.2 Behavioural activation, self-efficacy, and physical activity

Low mood, avoidance, and reduced activity can form a self-perpetuating cycle: Disengagement reduces opportunities for pleasure, mastery, and social support, thereby worsening depression and promoting further withdrawal (26, 27). Behavioural activation seeks to disrupt this cycle by systematically increasing activities that are consistent with personal values and capable of providing enjoyment or a sense of accomplishment (28). In social prescribing, shared decision-making, support with first attendance, and ongoing feedback may reduce apprehension and avoidance when entering an unfamiliar setting while increasing confidence in one’s ability to participate. A systematic review proposed positive social reinforcement, environmental enrichment, value-guided action, reduced avoidance, and enhanced social self-efficacy as potential pathways through which behavioural activation may promote social connection (29).

A pragmatic trial of peer companionship and qualitative research on social prescribing practice suggest that relational support can help some older adults overcome unfamiliar environments, mobility limitations, and low confidence (30, 31). Nevertheless, behavioural activation arising in social prescribing is usually an accompanying consequence of community participation rather than a deliberately administered psychotherapeutic technique. It differs from formal behavioural activation therapy, which typically includes structured activity scheduling, monitoring, and problem-solving components. An observed increase in activity can therefore indicate that an activation pathway may be operating; however, it cannot establish behavioural activation as the independent mechanism responsible for symptom change.

Physical activity is among the most common resources offered through social prescribing (32). A systematic review including randomised and quasi-randomised studies found signals of improvement in physical activity, but statistically significant findings occurred in only a minority of studies and were often limited to within-group changes. Substantial heterogeneity and risk of bias reduced confidence in the evidence (33). Longitudinal research in China has associated participation in community group exercise with improved depressive symptoms among middle-aged and older adults (34). Such findings support the plausibility of the pathway but do not directly evaluate the complete social prescribing process. Exercise may influence mood through neurotransmitter and hypothalamic–pituitary–adrenal pathways, improvements in self-efficacy, and positive social feedback (35). More broadly, structured increases in rewarding activities are also central to behavioural activation approaches for late-life depression (36). Late-life depression has also been associated with peripheral inflammatory markers, and exercise may modulate age-related low-grade inflammation (37, 38). As social prescribing studies rarely measure biomarkers alongside psychological mediators, these biological explanations remain hypotheses requiring direct testing.

### 4.3 Nature, arts, and social roles: combined effects and the restoration of meaning

Gardening, nature walks, and outdoor group programmes combine nature exposure with physical activity and social interaction. Attention restoration theory proposes that natural environments reduce demands on directed attention (39), while systematic reviews and meta-analyses suggest that nature-based interventions may improve depressive symptoms, cognition, and subjective wellbeing among older adults (40, 41). In assisted-living settings, nature connectedness has been associated with life satisfaction, feeling needed, and enthusiasm for life (42). Early reports from the Friends in Nature programme primarily support feasibility and acceptability (43). These findings are promising; however, they do not isolate the independent effect of nature exposure from movement, group interaction, staff attention, or selection into participation.

Retirement, bereavement, and declining function can disrupt social identity. Volunteering and community contribution may enhance psychological wellbeing by providing stable roles, broadening social networks, and strengthening the sense of being needed (44–46). Research on cultural social prescribing has identified immersion, psychological containment, relationship building, and personal growth as mechanisms perceived by participants (47). A broad review of arts and health and a meta-analysis of group-based arts interventions for late-life depression also support the potential value of arts participation (48, 49), although the type of activity, the level of participation, intensity, and characteristics of participants differ markedly across studies.

The common feature of these pathways is not simply an increase in activity but the restoration of meaningful daily structure and social identity. If social prescribing positions older adults only as passive recipients, it may secure short-term attendance without generating a durable sense of value. When participants can choose, assume a role, develop competence, and contribute to a group, an externally organised activity is more likely to become personally meaningful. A meta-analysis of 26 randomised controlled trials involving 3,892 older participants reported improvements in quality of life and reductions in loneliness (50). Nevertheless, variation in programme type, cultural context, intervention definition, and risk of bias limits the robustness and transferability of these estimates.

### 4.4 Nutrition and lifestyle: an adjunctive, not core, component

Higher-quality dietary patterns are associated with a lower risk of depression, and diet may influence mood through inflammation, oxidative stress, the gut–brain axis, and neurotrophic pathways (51, 52), with emerging neuroimmune research further supporting the biological plausibility of microbiota–brain signalling in mood regulation (53). A nationwide cross-sectional analysis using data from the 2018 Chinese Longitudinal Healthy Longevity Survey found that higher scores on a Chinese-adapted MIND diet were associated with fewer depressive and anxiety symptoms (54). These findings are relevant to the broader social determinants and lifestyle context of mental health, but they arise primarily from observational nutritional epidemiology rather than direct evaluations of social prescribing pathways. They therefore support biological and behavioural plausibility and not a claim that nutrition is an established mechanism of social prescribing.

Nutrition counselling, shared meals, and cooking groups may be included as adjunctive lifestyle resources within a broader social prescribing network, particularly when food access or social eating is relevant to the person’s goals. Their effectiveness is likely to depend on food availability, affordability, culinary skills, cultural preferences, social interaction, and sustained support. Nutrition should therefore be treated as a potential adjunct component rather than a core mechanism of social prescribing itself. Future studies should distinguish changes in nutritional knowledge from actual dietary behaviour and from the social effects of eating with others rather than allowing biological plausibility to substitute for evidence of intervention effectiveness.

## 5 Implementation requirements and priorities for optimisation

### 5.1 Population identification, risk stratification, and referral boundaries

Social prescribing should begin with the systematic identification of social needs. Primary care, chronic disease management, and routine health assessments for older adults offer opportunities to screen for loneliness, social isolation, functional limitation, caregiving strain, financial difficulty, and transport barriers, alongside assessment of anxiety, depression, and suicide risk (55). Screening scores can support but should not replace clinical judgement. For depression, the NICE uses PHQ-9 scores below 16 versus 16 or above as one indicator distinguishing less severe presentations from more severe presentations (56); the original PHQ-9 validation also supports a graded severity interpretation (57). For anxiety, a GAD-7 score of 15 or above is commonly used as a signal of severe symptom burden (58), but NICE recommends escalation primarily according to marked functional impairment, self-harm/suicide risk, complex comorbidity, self-neglect, or inadequate response to established treatment rather than a single score (59). A positive screen should therefore trigger clinical assessment, not automatic social prescribing referral.

Social prescribing is most appropriate when social risk is prominent, the person is willing to consider a community-based response, and immediate specialist treatment is not the overriding need. As an operational safety boundary, any current suicidal intent or significant self-harm risk, psychosis, mania, severe self-neglect, very marked functional impairment, or other evidence of acute/complex mental illness should prompt urgent or priority clinical assessment according to local pathways (56, 59). More severe depression (e.g., PHQ-9 ≥ 16 as a severity signal rather than a stand-alone referral rule), severe anxiety (e.g., GAD-7 ≥ 15), or substantial functional impairment should trigger a review for evidence-based psychological and/or pharmacological treatment; social prescribing may continue only as an adjunct when clinically appropriate. People with cognitive impairment, sensory loss, or mobility limitations may require carer involvement, accessible programmes, and transport support. Risk stratification is therefore essential both to protect safety and to prevent social prescribing from being presented as a universally applicable, low-cost alternative to established treatment.

### 5.2 Connector/link worker support, shared decision-making, and sustained engagement

Where medium or holistic models use a connector/link worker, that role provides a coordinating function between assessment, navigation, and continuing engagement. Realist studies indicate that professional competence, trusted relationships with healthcare teams and community organisations, clear role boundaries, and sustained support are important for embedding such models in routine care (60–62). In light or direct referral models, equivalent functions may be undertaken by the referring professional or programme staff rather than a dedicated link worker. Acceptance of a referral is not secured through a single act of consent; it develops through collaborative goal-setting, attention to concerns, and early experiences that demonstrate the feasibility and personal relevance of participation (63).

A high-quality referral pathway includes at least four functions: Identifying needs and risks; agreeing on goals around what matters most to the individual; matching the person with an accessible and meaningful resource; and monitoring participation, difficulties, and outcomes, with feedback to the clinical team. Simply providing an activity list is unlikely to change behaviour. For older adults with social anxiety, longstanding avoidance, or functional limitation, accompanied first attendance, a trial period, and adjustment of the plan may determine whether a referral translates into meaningful participation.

### 5.3 Community capacity, cross-sector collaboration, and equity

Social prescribing redirects demand towards community resources, but it does not create those resources. Sustainability depends on programme capacity, trained staff, suitable venues, transport, affordability, safety, and cultural fit. Models of social prescribing also differ: Some emphasise clinical referral and lifestyle management, whereas others prioritise social determinants, empowerment, or community development (64). Evaluation should therefore extend beyond referral counts to examine resource coverage, equity, completion, and the burden placed on community organisations (65).

Implementation studies from the United Kingdom and Portugal identify unstable funding, workforce shortages, weak cross-sector communication, disconnected data systems, and inadequate support after referral as recurrent barriers (66, 67). These constraints may be more pronounced in resource-limited settings. In China, community health centres, ageing service stations, universities for older people, public cultural and sports facilities, and social organisations constitute a potentially substantial asset base. However, fragmented governance, poor visibility of available resources, and shortages of trained navigators may leave resources available but difficult to access. Local implementation should begin with community asset mapping and clear eligibility and service standards, followed by gradual expansion that does not exceed the capacity of community providers.

### 5.4 Digital support and closed-loop evaluation

Digital platforms may support community resource directories, referral tracking, reminders, and collection of patient-reported outcomes. Stakeholder research suggests that digital technologies could improve matching and access, but implementation is constrained by funding, interoperability, privacy, digital literacy, and limited evidence (68). Qualitative research with older adults also identifies physical limitations, transport difficulties, and digital exclusion as barriers, while personalised support, accessible settings, and assistance with technology can facilitate engagement (69).

Artificial intelligence may assist with risk identification or resource recommendation, but it should not replace the relational judgement of a trained link worker. A systematic review of artificial intelligence-based approaches to loneliness and social connectedness in older adults reported potential benefits but also highlighted small samples, technological heterogeneity, and limited long-term evidence (70). Digital models should therefore retain telephone, paper-based, and face-to-face options and, with the older person’s consent, allow assistance from relatives or carers so that efficiency measures do not widen inequalities.

Evaluation should cover structure, process, and outcomes. Structural indicators include the availability of resources, link worker capacity, and accessibility. Process indicators include referral timeliness, first-contact rates, actual uptake, continued participation, and reasons for withdrawal. Outcomes should encompass anxiety, depression, loneliness, quality of life, functioning, and service use. As social prescribing is a complex intervention, evaluation should combine outcome measurement with process evaluation, realist methods, and implementation outcomes to explain how and why a programme works for particular populations and settings, rather than relying only on pre–post changes in rating scales (13, 71).

## 6 Discussion

Taken as a whole, social prescribing is conceptually well aligned with the complex aetiology of anxiety and depression in later life. It brings loneliness, reduced activity, loss of role, and inadequate community support into the scope of intervention and may provide continuity beyond biomedical treatment (8, 9). Current studies have shown encouraging signals for loneliness, quality of life, social participation, and selected depressive outcomes (20, 33, 50). Implementation research also consistently identifies shared decision-making, link worker support, and accessible community resources as important conditions for benefit (60–67). The principal value of social prescribing may therefore lie in its capacity to integrate clinical and community responses rather than in the specific therapeutic effect of any single activity. This emphasis is consistent with broader chronic care models that prioritise patient-centred coordination, community support, and integration of physical and mental healthcare (72).

The certainty of evidence nevertheless remains limited and does not support unqualified large-scale implementation without population stratification and adequate service infrastructure. Few randomised trials specifically enrol older adults with clinically diagnosed anxiety or depressive disorders; many focus instead on loneliness, multimorbidity, subthreshold symptoms, or general community populations. The activities included, level of support, and referral pathways vary substantially, meaning that interventions described by the same label may be operationally very different. Outcome measures and follow-up periods are inconsistent, while attrition and self-selection are common. People who agree to community participation may already have greater motivation, confidence, or functional capacity. In addition, several mechanistic claims are extrapolated from research on exercise, nature, arts, or nutrition and cannot establish a causal effect of the complete social prescribing pathway. The present review also has methodological limitations. As a narrative synthesis, it was designed to integrate heterogeneous direct, pathway, and implementation evidence rather than to provide an exhaustive systematic estimate of intervention effects. The structured search may not have captured all relevant literature, and design-specific risk-of-bias tools were not applied across all source types. The conclusions should therefore be interpreted as a critical conceptual synthesis of the available evidence rather than as a definitive estimate of intervention effectiveness.

These uncertainties do not negate the potential value of social prescribing; they require more precise research questions. Future studies should move from asking whether social prescribing works to asking for whom, through which mechanisms, and under what conditions it works. Trials should clearly report the referral source, link worker contact intensity, activity dose, community context, comparators, and co-interventions and should distinguish clinical outcomes from mediating variables and implementation outcomes. Process evaluation and mediation analysis embedded within randomised studies may help identify core functions, adaptable components, and the populations most likely to benefit (13, 71). Mechanistic research should measure loneliness, social network change, activity, self-efficacy, and functioning together. Biomarkers should be included only when supported by an explicit causal hypothesis rather than as a substitute for examining failures in referral, access, or engagement.

Local adaptation should avoid uncritical replication of any single British model, including the link worker-intensive holistic model. Primary care organisation, family caregiving, and community governance differ across countries, and lower-intensity light or direct referral models may be appropriate where needs are specific and access barriers are limited (10, 11). In China, social prescribing could be integrated with health assessments for older adults, family doctor services, chronic disease management, and health and social care integration. Initial programmes should focus on people with clearly identified social risks, mild-to-moderate symptoms, and community resources capable of responding. Community organisations require stable funding, training, and data support. Evaluation should pay particular attention to access among rural residents, people with low incomes, those living alone, people with disabilities, and older adults with limited digital capability. Social prescribing is likely to reduce unmet needs only when community capacity is developed alongside clinical referral rather than when responsibility is simply transferred out of healthcare.

The most defensible current conclusion is that social prescribing is a theoretically grounded complex service model with emerging empirical support. It may improve loneliness, social participation, quality of life, and selected emotional outcomes, but certainty regarding its effects on clinically diagnosed anxiety and depression remains limited. Its success depends on coordination across needs assessment, shared decision-making, sustained relational support, community capacity, and feedback rather than on the completion of a single referral to a community activity.

## 7 Conclusion

By connecting health services with community resources through models of varying intensity, social prescribing may address social and behavioural determinants of anxiety and depression in older adults and can be positioned as an adjunct to evidence-based pharmacological and psychological care. Current evidence suggests potential benefits for loneliness, social participation, quality of life, and selected depressive outcomes, but direct clinical evidence, mechanistic validation, long-term sustainability, and cost-effectiveness remain insufficient. Future research should define referral and escalation boundaries, report the intensity and functions of each social prescribing model, test proposed mediators directly, and evaluate locally adapted approaches centred on meaningful resource matching, proportionate relational support, equitable access, community capacity, and closed-loop outcome assessment.
